# Clinical Utility of Emergency Capsule Endoscopy for Diagnosing the Source and Nature of Ongoing Overt Obscure Gastrointestinal Bleeding

**DOI:** 10.1155/2019/5496242

**Published:** 2019-12-01

**Authors:** Sumio Iio, Shiro Oka, Shinji Tanaka, Akiyoshi Tsuboi, Ichiro Otani, Sayoko Kunihara, Kazuaki Chayama

**Affiliations:** ^1^Department of Gastroenterology and Metabolism, Hiroshima University Hospital, Hiroshima, Japan; ^2^Department of Endoscopy, Hiroshima University Hospital, Hiroshima, Japan

## Abstract

**Background and Aims:**

In patients with ongoing overt obscure gastrointestinal bleeding (OGIB), prompt detection of the bleeding source is crucial to treatment success. However, there is no consensus on the optimal timing of diagnostic capsule endoscopy (CE). We investigated the clinical utility of emergency CE for detecting the source of ongoing overt OGIB.

**Methods:**

We retrospectively evaluated 146 consecutive patients who, between February 2009 and July 2018, underwent emergency CE at Hiroshima University Hospital to detect the source of ongoing overt OGIB. Patients with a bleeding source located outside the small bowel were excluded. The remaining 127 patients were stratified according to the timing of CE relative to the onset of bleeding: patients in group A (*n* = 15, 12 men; mean age: 75 years; age range: 62–83 years) underwent CE within 48 hours of bleeding onset, whereas patients in group B (*n* = 112, 73 men; mean age: 65 years; age range: 17–88 years) underwent CE at >48 hours after bleeding onset. All patients underwent double-balloon endoscopy, and the final diagnosis was compared against the CE findings.

**Results:**

The CE lesion detection rate was significantly higher in group A (12/15 patients, 80%) than in group B (53/112 patients, 47%) (*p* = 0.0174). There was no significant difference between the two groups regarding the patients' background characteristics. Vascular lesions were the most frequent finding in both groups. The diagnostic concordance rate between emergency CE and double-balloon endoscopy was 100% in group A and 92.9% in group B. Rebleeding after endoscopic treatment was confirmed in only one patient in group B.

**Conclusions:**

Emergency CE represents a useful diagnostic modality in patients with ongoing overt OGIB, potentially improving detection rates and reducing rebleeding risk.

## 1. Introduction

Obscure gastrointestinal bleeding (OGIB) is broadly categorized into overt OGIB and occult OGIB based on whether bleeding is clinically evident [1]. Overt OGIB is further categorized as ongoing or previous, based on whether bleeding is immediately visible upon observation. The definition of OGIB varies across studies and the diagnosis and treatment have evolved to reflect the advancements in imaging technologies. In patients with ongoing overt OGIB, it is necessary to detect the bleeding source as soon as possible in order to determine the optimal treatment plan and initiate treatment in a timely manner. The clinical practice guidelines issued by the Japanese Gastroenterological Endoscopy Society (JGES) and other reports recommend capsule endoscopy (CE), a minimally invasive and safe procedure, as a useful diagnostic modality for OGIB [2–7]. Ongoing overt OGIB is often caused by severe lesions, which carry a high risk of recurrent bleeding. Therefore, emergency CE may facilitate early diagnosis and thus timely and adequate treatment of ongoing overt OGIB. The JGES guidelines recommend that examinations be carried out immediately or as soon as possible; however, the optimal timing of CE in ongoing overt OGIB remains unclear [2]. In this study, we investigated the clinical utility of emergency CE for detecting the source of ongoing overt OGIB.

## 2. Materials and Methods

### 2.1. Patients

This was a retrospective review of medical records maintained in our hospital's database. We identified 146 consecutive patients (85 males; mean age: 68 years) who, between February 2009 and July 2018, underwent CE at Hiroshima University Hospital for ongoing overt OGIB (6% of patients who underwent CE during the study period). We defined ongoing overt OGIB as continuous overt bleeding with no bleeding source identified on esophagogastroduodenoscopy and colonoscopy. Before CE, all patients underwent transabdominal ultrasonography and/or abdominal computed tomography to rule out gastrointestinal tract stenosis and small-bowel disease. However, the bleeding source remained obscure, and the patients were indicated for CE.

### 2.2. Study Design

**Figure 1:**
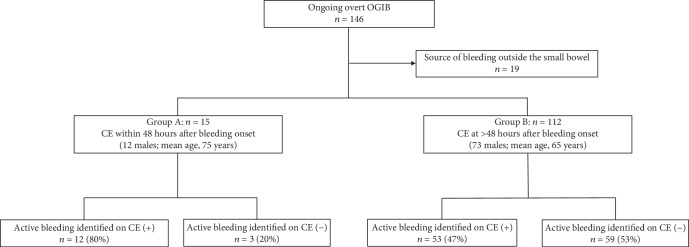
Flow chart of patient enrolment, allocation, and analysis. Consecutive patients with ongoing overt OGIB were stratified according to the timing of CE relative to the onset of bleeding. All patients underwent double-balloon endoscopy, and the final diagnosis was compared against the CE findings. Abbreviations: CE, capsule endoscopy; OGIB, obscure gastrointestinal bleeding.

A flow chart of patient enrollment, allocation, and analysis is illustrated in Figure 1. Patients with a bleeding source located outside the small bowel were excluded. The remaining 127 patients were stratified according to the timing of CE relative to the onset of bleeding: patients in group A (*n* = 15; 12 males; mean age, 75 years; age range, 62–83 years) underwent CE within 48 hours of bleeding onset, whereas patients in group B (*n* = 112; 73 males; mean age, 65 years; age range, 17–88 years) underwent CE at >48 hours after bleeding onset. After CE, all patients underwent double-balloon endoscopy (DBE). Retrograde and/or antegrade DBE were performed and the entire small-bowel was observed. The final diagnosis obtained via DBE was compared against the CE findings. The effect of CE timing on the concordance between CE and DBE was examined.

This study was conducted in accordance with the Declaration of Helsinki. The patients were informed of the risks and benefits of CE at the time of the procedure, and each provided written informed consent for the use of their deidentified data for research purposes. The study protocol was approved by the Institutional Review Board of Hiroshima University Hospital (approval number: E-943).

### 2.3. CE Procedure

CE was performed using a PillCam™ SB2 or SB3 video capsule (Covidien, Mansfield, MA). Sensor arrays were attached to the patient's abdomen, and a data recorder was attached to a belt fitted around the waist. The patient swallowed the capsule with a solution of dimethicone after a 12 h overnight fast. Although the patients were instructed to swallow the capsule in the sitting position, they were allowed to resume their normal activities immediately thereafter. After 8 hours, the sensor arrays and the recording device were removed. Images were analyzed using the Rapid Reader 6.5 software running on a RAPID 8 workstation (Covidien). The CE digital image stream was reviewed and interpreted independently by two experienced professionals who had reviewed images from >200 patients. Diagnoses were reached by consensus.

### 2.4. Data Collection and Evaluation

All data were extracted from the medical records maintained by our hospital. Ongoing overt OGIB was defined as persistent overt bleeding with unexplained cause after endoscopic examination of the upper and lower gastrointestinal tract. We evaluated the clinical characteristics, treatment method for small-bowel lesions, and rebleeding rate after treatment in groups A and B. The following clinical characteristics were evaluated: sex, age, concomitant disease, medication, duration of disease, hemoglobin level (g/dL), need for blood transfusion, types of hemostasis employed, and endoscopic findings (capsule delivery to the small bowel, presence of angioectasia, and presence of bleeding).

### 2.5. Statistical Analysis

The patients were stratified first based on the timing of CE (at ≤48 or >48 hours after the onset of bleeding) and then based on the results CE (positive or negative). Between-group differences were evaluated using Student's *t*-test for quantitative data and the chi-squared test for categorical data. Yates' correction or Fisher's exact test was used, as appropriate. All tests were two-sided, and *p* values < 0.05 were considered to indicate statistical significance. Survival was analyzed using the Kaplan-Meier method, and the differences between subgroups were examined using the log-rank test. All analyses were performed using JMP version 13 (SAS Institute Inc., Cary, NC).

## 3. Results and Discussion

The small-bowel lesion detection rate on CE was significantly higher in group A than in group B (12/15 patients, 80% vs. 53/112 patients, 47%; *p* = 0.0174).  Table 1 summarizes the characteristics of the two patient groups. Both groups contained more men than women, with a similar ratio (group A: 37% women, 5/15; group B: 42% women, 47/112; *p* = 0.5189) and with no significant difference in age (group A: 80% aged ≥65 years, 12/15; group B: 54% aged ≥65 years, 67/112; *p* = 0.0837). There was no significant difference in the prevalence or nature of concomitant diseases, in the hemoglobin level, in transfusion requirements, or in the use of nonsteroidal anti-inflammatory drugs (NSAIDs) or antiplatelet drugs. Furthermore, vascular lesions (especially angioectasias) were the most frequent findings in both groups.

Table 2 provides a summary of the treatment methods for small-bowel lesions. Endoscopic hemostasis was performed for all vascular lesions, except in one patient from group B who had an ileal arteriovenous fistula. Endoscopic hemostasis was difficult in this case, and surgery was required. Among the patients in group A, endoscopic hemostasis was performed for NSAID-induced ulceration, whereas nonspecific ulcerations were treated using medication alone. Among the patients in Group B, interventional radiology was performed for one nonspecific ulceration, and endoscopic hemostasis or medication were utilized in the remaining cases. In both groups, neoplastic lesions and Meckel's diverticula were treated with surgery or followed up without treatment, as clinically indicated. Rebleeding after treatment was confirmed in only one patient from group B (2%, 1/53), in whom the culprit lesion was an ileal artery fistula (Table 3).

There was high diagnostic concordance between CE and DBE for the detection of small-bowel lesions (Table 4). Only five patients with negative CE findings had positive DBE findings (false-negative CE results), whereas three patients with positive CE findings had negative DBE findings (false-negative CE results). For each imaging modality (CE and DBE), detection of a lesion was considered a positive result. Considering the DBE diagnosis as a reference, CE had a sensitivity of 92% (57/62) and specificity of 95% (62/65). The diagnostic concordance rate between CE and DBE was 94% (119/127) for positive findings. The sensitivity of CE was 100% (12/12) in group A and 90% (45/50) in group B, whereas specificity was 100% (12/12) in group A and 95% (59/62) in group B. The diagnostic concordance rate between CE and DBE was 100% (15/15) in group A and 93% (104/112) in group B.

The clinical characteristics of cases involving false-negative CE results are summarized in Table 5. Four of the five patients with negative CE findings were diagnosed as having angioectasia (based on the DBE findings) and were treated with endoscopic hemostasis. The remaining patient had an NSAID-induced ulceration that required treatment with a mucosal protectant after stopping NSAID therapy.

A recent meta-analysis reported that CE and DBE have similarly high diagnostic yields for small bowel lesions [8]. The JGES issued clinical practice guidelines that provide a clear diagnostic strategy for OGIB but not for ongoing overt OGIB, especially regarding the optimal timing of CE [2]. On the other hand, OGIB has become the most frequent indication for CE [9, 10]. Importantly, CE has been reported to provide a significantly higher diagnostic yield for ongoing overt OGIB than for occult OGIB, with the highest diagnostic yield (up to 92%) achieved when it is performed as close as possible to the bleeding episode [11–14]. However, previous studies have focused mainly on patients with persistent or recurrent OGIB rather than ongoing OGIB, and some studies have reported on the timing of CE for ongoing overt OGIB [13, 15–17].

In everyday clinical practice, CE is generally performed after gastroscopy and/or colonoscopy. However, since only major referral hospitals are equipped to perform both CE and DBE in Japan, patients rarely undergo CE within 24 hours after the onset of bleeding. Therefore, in our study, emergency CE was defined as CE performed within 48 hours after the onset of bleeding. We found that the timing of CE (before/after the 48-hour threshold) had a significant impact on the detection rate of bleeding sources in the small bowel of patients with ongoing overt OGIB. Specifically, the detection rate was significantly higher in group A (CE performed within 48 hours) than in group B (CE performed at >48 hours). Furthermore, conducting CE within 48 hours of bleeding onset improved the diagnostic concordance rate between CE and DBE.

In our study, vascular lesions were the most frequent bleeding sources of ongoing overt OGIB, but such lesions were highly amenable to endoscopic hemostasis, which is in agreement with previous observations [18, 19]. Rebleeding was confirmed in only one patient, in whom the culprit lesion was an ileal artery fistula. The rebleeding was confirmed at 2 months after endoscopic hemostasis. As the hemorrhage could not be controlled with repeat endoscopic hemostasis, the patient underwent embolization by an interventional radiologist. However, this procedure was also unsuccessful, and the patient was indicated for emergency surgery.

Although we did not find any association between the timing of CE and the rate of rebleeding, it should be noted that this effect might have been masked by the fact that only one patient had rebleeding after treatment. Emergency CE is expected to help reduce the risk of rebleeding. We previously reported good outcomes in patients who undergo total enteroscopy and receive proper treatment for bleeding sources located in the small bowel [20]. Furthermore, in a study of OGIB patients with ulcerative lesions, Aoki et al. [21] reported that the rebleeding rate was lower for single ulcerations than for multiple ulcerations. It should also be noted that small-bowel vascular lesions have a higher rate of rebleeding than other types of lesions, even after endoscopic interventions [22]. Yung et al. [23] reported that negative CE findings generally reflect a low risk of subsequent rebleeding, and such patients could be safely managed with watchful waiting. Other studies reported a 0% rebleeding rate of overt and occult OGIB over a 12-month follow-up period after CE with negative results [24, 25]. Niikura et al. reported that female gender, liver cirrhosis, warfarin use, positive CE findings, and overt bleeding were significant predictors of rebleeding [26]. Our present findings are in agreement with these previous observations, revealing a 100% diagnostic concordance rate between CE and DBE in patients who underwent emergency CE (group A; 15/15), thus supporting the contribution of emergency CE to reducing the risk of rebleeding.

In this study, CE detected the source of bleeding in the small bowel in 45% (57/127) of patients with ongoing overt OGIB. The diagnostic concordance rates between CE and DBE were high in both groups (group A: 100%; Group B: 93%). Some studies have reported that performing CE within 24–72 hours from the onset of overt OGIB results in a diagnostic yield higher than 60% [14–16]. Apostolopoulos et al. [14] reported that emergency CE (within 48 hours) revealed active bleeding in 34 patients with negative findings after upper and lower gastrointestinal endoscopies, providing a diagnostic yield of 91.9%. Taken together, our present results and these previous observations support diagnostic strategies that involve performing CE as soon as possible after the onset of overt OGIB, as such strategies are likely to improve the chance of detecting the bleeding source. Lecleire et al. [15] reported that emergency CE (within 24–48 hours) for severe overt OGIB identified the bleeding lesions in 67% of patients. We presently found 100% sensitivity and specificity for emergency CE in patients with ongoing overt OGIB. Thus, we believe that performing CE within 48 hours of bleeding onset contributes to the identification of bleeding sources and facilitates timely initiation of adequate treatment.

Our study has several limitations. First, this was a retrospective analysis, and thus selection bias could not be excluded. Second, the sample size was relatively small and only one patient experienced rebleeding. Third, the data were obtained from a single center, and our observation period was relatively short. Therefore, a large-scale study is needed to address these limitations.

## 4. Conclusions

Emergency CE is useful for identifying and diagnosing bleeding sources in the small bowel of patients with ongoing overt OGIB. Patients with positive CE findings can be indicated for DBE or another suitable treatment, whereas patients with negative findings on emergency CE may be observed without treatment. Although large-scale studies are warranted to confirm our present observations, we believe these findings represent a step towards establishing clinical practice guidelines regarding the optimal timing of CE in patients with ongoing overt OGIB.

## Figures and Tables

**Table 1:** Patient and lesion characteristics at the time of capsule endoscopy for identifying the source of ongoing overt obscure gastrointestinal bleeding.

Variables	Group		*P* value
	A, *n* = 15, CE at ≤48 h	B, *n* = 112, CE at >48 h	
Sex			
Male	10 (67)	65 (58)	0.5189
Female	5 (37)	47 (42)	
Age category			
<65 years	3 (20)	45 (46)	0.0837
≥65 years	12 (80)	67 (54)	
Concomitant disease			
Cardiovascular disease	3 (20)	9 (10)	0.0650
Chronic renal failure	1 (7)	4 (7)	
Chronic liver disease	0 (0)	2 (2)	
Cerebrovascular disease	1 (7)	1 (2)	
Medication			
Antiplatelet drugs	2 (13)	15 (13)	0.9949
NSAIDs	0 (0)	11 (10)	0.0871
Hb level (g/dL)	9.2	7.1	0.7049
Transfusion	3 (20)	30 (29)	0.5736
Lesion type	12 (80)	53 (47)	0.0174
Vascular lesion	5 (33)	24 (21)	0.3023
Ulcerative lesion	2 (13)	20 (18)	0.6637
Neoplastic lesion	4 (27)	8 (7)	0.0356
Meckel's diverticulum	1 (7)	1 (1)	0.1801

The patients were stratified according to the timing of CE relative to the onset of bleeding. Data are shown as frequency (percentage) or mean, as appropriate. Abbreviations: CE, capsule endoscopy; Hb, hemoglobin; NSAID, nonsteroidal anti-inflammatory drug.

**Table 2:** Treatment methods for small-bowel lesions causing ongoing overt obscure gastrointestinal bleeding.

	*N* (%)
Group A, *n* = 15, CE at ≤48 h	
Vascular lesion	5 (33)
Endoscopic hemostasis	5 (33)
Ulcerative lesion	2 (13)
Endoscopic hemostasis	1 (7)
Medication	1 (7)
Neoplastic lesion	4 (27)
Surgery	2 (13)
No treatment	2 (13)
Meckel's diverticulum	1 (7)
No treatment	1 (7)
Group B, *n* = 112, CE at >48 h	
Vascular lesion	24 (21)
Endoscopic hemostasis	23 (20)
Surgery	1 (1)
Ulcerative lesion	20 (18)
Medication	10 (9)
Endoscopic hemostasis	9 (8)
Interventional radiology	1 (1)
Neoplastic lesion	8 (7)
Surgery	6 (5)
No treatment	2 (2)
Meckel's diverticulum	1 (1)
Surgery	1 (1)

The patients were stratified according to the timing of CE relative to the onset of bleeding. Abbreviations: CE, capsule endoscopy.

**Table 3:** Rebleeding rates stratified according to the initial treatment for ongoing overt obscure gastrointestinal bleeding.

Rebleeding rate	
Group A, *n* = 15, CE at ≤48 h	
0/12 (0%)	
Endoscopic hemostasis	6 (50)
Surgery	2 (17)
Medication	1 (8)
No treatment	3 (25)
Group B, *n* = 112, CE at >48 h	
1/53 (2%)	
Endoscopic hemostasis	32 (60)
Medication	10 (20)
Interventional radiology	1 (2)
Surgery	8 (14)
No treatment	2 (4)

The patients were stratified according to the timing of CE relative to the onset of bleeding. Data are shown as frequency (percentage). Abbreviations: CE, capsule endoscopy.

**Table 4:** Diagnostic concordance rate between CE and DBE for small-bowel lesions causing ongoing overt obscure gastrointestinal bleeding.

CE findings	DBE findings		Total
	Positive	Negative	
Positive	57	3	60
Negative	5	62	67
Total	62	65	127

All patients underwent DBE, and the final diagnosis was compared against the CE findings. Emergency CE had a sensitivity of 92% (57/62) and specificity of 95% (62/65), with a diagnostic concordance rate of 94% (119/127) for positive findings. Abbreviations: CE, capsule endoscopy; DBE, double-balloon endoscopy.

**Table 5:** Clinical characteristics of cases with false-negative results on emergency capsule endoscopy to detect the source of ongoing overt obscure gastrointestinal bleeding.

Case no.	Sex	Age (years)	Complication	Antiplatelet drugs	NSAIDs	Diagnosis (Yano-Yamamoto's classification)	Location	Treatment
1	M	83	Liver cirrhosis	+	—	Angioectasia (type 1b)	Ileum	Endoscopic hemostasis
2	M	55	—	—	—	Angioectasia (type 1b)	Jejunum	Endoscopic hemostasis
3	M	51	Cardiovascular disease	+	—	Angioectasia (type 1b)	Ileum	Endoscopic hemostasis
4	F	75	Low back pain	—	+	NSAID-induced ulceration	Ileum	Medication
5	F	65	Liver cirrhosis	—	—	Angioectasia (type 1a)	Jejunum	Endoscopic hemostasis

Abbreviations: F—female; M—male; NSAID—nonsteroidal anti-inflammatory drug.

## Data Availability

The data used to support the findings of this study are included within the article.
